# Clinical Influence of Micromorphological Structure of Dental Implant Bone Drills

**DOI:** 10.1155/2018/8143962

**Published:** 2018-06-06

**Authors:** Gaetano Marenzi, Josè Camilla Sammartino, Giuseppe Quaremba, Vincenzo Graziano, Andrea El Hassanin, Med Erda Qorri, Gilberto Sammartino, Vincenzo Iorio-Siciliano

**Affiliations:** ^1^Division of Oral Surgery, Department of Neurosciences, Reproductive and Odontostomatological Sciences, University “Federico II”, Via Pansini 5, Naples, Italy; ^2^Department of Biology and Biotechnology, University “L. Spallanzani”, Via Ferrata 9, Pavia, Italy; ^3^Department of Industrial Engineering, University of Naples “Federico II”, Via Claudio 21, Naples, Italy; ^4^Department of Advanced Biomedical Sciences, University “Federico II”, Via Pansini 5, Naples, Italy; ^5^Department of Chemical, Materials and Production Engineering, University “Federico II”, Piazzale Tecchio 80, Naples, Italy; ^6^Division of Oral and Maxillofacial Surgery, Department of Dentistry, Albanian University. Str. Durres, Tirana, Albania; ^7^Department of Periodontology, University of Catanzaro “Magna Graecia”, Viale Europa, Loc. Germaneto, Catanzaro, Italy

## Abstract

**Background:**

Considerations about heat generation, wear, and corrosion due to some macrostructural bur components (e.g., cutting lips, rake angle, flute, and helix angle) have been widely reported. However, little is known about how the microstructural components of the implant drill surface can influence the implant drill lifetime and clinical performance.

**Aim:**

To investigate accurately the surface morphology of surgical bone drill, by means of multivariate and multidimensional statistical analysis, in order to assess roughness parameters able to predict the evolution of tribological phenomena linked to heat development, wear, and corrosion occurring in clinical use.

**Materials and Methods:**

The surfaces of implant drills approximately 2.0mm in diameter made by five manufacturers were examined by means of confocal microscope with white light laser interferometry, obtaining several surface roughness parameters. Statistical multivariate analysis based on discriminant analysis showed, for each cut-off, the parameters which discriminate the manufacturers.

**Results:**

The microstructural parameters used by discriminant analysis evidenced several differences in terms of drill surface roughness between the five manufacturers.

**Conclusions:**

The observed surface roughness difference of drills is able to predict a different durability and clinical performance especially in heat generation and wear onset.

## 1. Introduction

Minimally traumatic preparation of the implant socket is critical for predictability and enhanced osseointegration [1]. The preservation of the original bony microstructure, especially of the cancellous bone and its high osteogenic potency, will benefit the bone healing process [2]. Although osteotomy site preparation has been studied for decades, there remains a remarkable lack of consensus on what constitutes an optimal method to cut bone. Major problems faced during bone drilling were thermal necrosis, bur deformation, and the generation of microcracks on the inner surface of the drilled holes that can detrimentally affect osteosynthesis and healing [3, 4]. In clinical practice, to perform osteotomies for dental implant placement, rotary cutting instruments (burs) are used and efforts have been made to develop implant drills with improved mechanical proprieties [5–10]. Many aspects can significantly affect their cutting efficiency and durability: design, diameter, composition and surface treatment, mechanical properties, drill rotational speed, axial drilling forces, cooling, and the sterilization process [1, 5, 10–15]. Some authors evidenced also the drill corrosion as a potential key factor in determining lifespan of the implant burs [1, 12, 16, 17].

Many of these drilling parameters also play a primary role in controlling the temperature generated during the osteotomy [1, 5, 11–14, 18, 19]. Their bone damage is related to the magnitude of the temperature elevation and the time during which the tissue is subjected to damaging temperature, identified as 47°C for 1 min [1, 9, 10, 13]. The heat generated during the implant site preparation is related to the surface cutting power [2, 15] and hence to the manufacturer's precision [2, 3]. Magnification of the cutting tip of the drills showed many differences in the manufacturing of the drills. The two-fluted drill was correlated to less heat generation whereas the three-fluted drill showed somewhat favorable cutting efficiency [2]. Heat generation, as evidenced by Matthews and Hirsch [20], can be also influenced by the manufacturer's precision (sharpness of the cutting tool) [15] and the surface deformation and roughness showed by the worn burs that cause a more significant and continuous temperature rise than new burs [5, 12, 14, 21]. No clear suggestion is made on the number of times that the drill can be used repeatedly until it becomes blunt and ineffective, producing a significant increase in temperature [11, 13]. Since the sharpness of the drill bit is one of the most important factors when considering the temperature increase, to minimize this surgical trauma well-sharpened drills are recommended [13, 22–24]. In a previous study SEM analysis evidenced manufacturing defects in new drills which increased in number and deteriorated with use [9, 14]. These defects influence the cutting efficiency, favoring heat and bone microcrack generation, and reduce the time when the reused drills can be considered sharp enough. Considerations about heat generation and wear with some macrostructural bur components (e.g., cutting lips, rake angle, flute, and helix angle) have been extensively reported. Also microstructural components of the bur surface can influence heat generation and wear.

The aim of this study was to investigate accurately the surface morphology of surgical bone drills through the confocal microscopy and by using multivariate and multidimensional statistical analysis, in order to assess roughness parameters able to predict the evolution of tribological phenomena linked to heat development, wear, and corrosion phenomena occurring in clinical use.

## 2. Material and Methods

Implant drills approximately 2.0mm in diameter were selected because they usually represent one of the first bone drills to be used for implant site preparation, drilling both cortical and cancellous bone.

The following implant bone drills made by five manufacturers were analyzed:

1. Straumann (Straumann AG, Waldenburg, Switzerland) 2.2mm in diameter (for short, A)
2. Nobel Biocare (Nobel Biocare AB, Goteborg, Sweden) 2.0mm in diameter (for short, B)
3. Xive Implant System (Friadent GmbH, Mannheim, Germany) 2.0mm in diameter (for short, C)
4. Global D (French) 2.5mm in diameter (for short, D)
5. Sweden & Martina** (**Padova, Italy**)** 2.5mm in diameter (for short, E)

The rugosimetric survey was carried out through a Leica DCM 3D confocal microscope with white light laser interferometry, which makes it possible to study the surface finish of the drills in high resolution. More specifically, confocal microscopy allows the reconstruction through several optical sections, without any physical contact with parts, of complex 3D surfaces that cannot be analyzed otherwise [25]. Using, for example, contact profilometers, filtering was then carried out, based on the choice of cut-off, in order to separate the roughness profile from the waviness profile (or geometric shape). On the basis of preliminary performed measurements and extraction of primary profiles, according to the international standard ISO 4287 [26], since the acquired profile seemed to be quite periodic, P_sm_ (i.e., spacing parameters obtained by the primary profile) was chosen as the parameter to determine the appropriate value of the cut-off length. Moreover, since, in our case, the above-mentioned parameter, P_sm_, is very close to the boundary value of 0.04mm, which separates two different cut-off values applicable, i.e., 0.08mm and 0.25mm, both cut-off values were taken into account in determining the roughness and waviness profiles and relative parameters. The filtering operation was carried out with a robust Gaussian filter. For each manufacturer, three bone drills were employed. For each filtering operation, three profiles parallel to the acquisition direction and three perpendicular to the acquisition direction were extracted. In this way, the parameters prescribed by ISO 4287-1997 [26] were obtained, accordingly described below.

The distortions due to the ends of the profile and the finite length of the profile were minimized by removing a portion of profile at the beginning (run-up) and at the end of profile (run-down) and by applying a minimum number of cut-offs in the measured profile (evaluation length), namely, three.

Surface texture can be described in quantitative terms by means of a certain number of parameters. All of these parameters represent different aspects of the surface, such as roughness, waviness, and shape. In order to predict the behavior of a component during its normal use, it is necessary to quantify the surface characteristics. This is possible through the parameters mentioned below. They can be classified into amplitude, spacing, and hybrid parameters, in particular, (i) amplitude parameters: R_p_, R_v_, R_z_, R_c_, R_t_, R_a_, R_q_, R_sk_, R_ku_ [26]; (ii) material ratio parameters: R_mr_ (*c*= 1*μ*m below the highest peak), R_dc_ (p = 5%, q = 95%) [26]; (iii) spacing parameters: primary profile, PS_m_ [27]; (iv) hybrid parameters: R_sk_, R_ku_.

The best known and commonly used parameter is R_a_, defined as the arithmetical mean of the absolute values of the profile deviations from the mean line of the roughness profile [26]. This is an amplitude parameter that is useful for preliminary analysis of the surface finish. R_a_ is used as a global evaluation of the roughness amplitude on a profile. It does not say anything on the spatial frequency of the irregularities or the shape of the profile. R_a_ is meaningful for random surface roughness (stochastic) machined with tools that do not leave marks on the surface, such as sand blasting, milling, and polishing. Since the aim of the paper was to assess surface parameters that are related to phenomena such as heat generation, wear, and corrosion, our attention also turned to hybrid parameters: R_sk_ and R_ku_. R_sk_ parameter was chosen because it represents the profile asymmetry with respect to the mean line. When it is positive, the profile is more pronounced toward the peaks. By contrast, when it is negative the profile is more pronounced toward the valleys. That said, the greater the distance from zero, the more asymmetric the profile. The second parameter, R_ku_, was chosen because it is an index of the shape of the peaks and more specifically their “tailness”; i.e., the higher the value, the more pronounced the peaks.

These parameters, according to the consideration made also by Karl Niklas Hansson and Stig Hansson in their work [28], represent in our case the best indicators for the phenomena involved. In a first step, multifactorial statistical analysis was performed starting from a hypothesis-free perspective. This implies that no “outcome” variables based on clinical experience were selected because the aim was to identify potential associations that might have been overlooked before.

Therefore, all variables were tried in the analysis. The approach was performed by using discriminant analysis (DA). The aim is to statistically distinguish between the five groups of makers. To distinguish between the groups, a collection of discriminating variables that measure the characteristics on which the groups are expected to differ were selected (i.e., all the above-mentioned parameters). The mathematical objective of DA is to weight and linearly combine the discriminating variables in some fashion so that the groups are forced to be as statistically distinct as possible. The statistical theory of DA assumes that the discriminating variables have a multivariate normal distribution and that they have equal variance-covariance matrices within each group. In practice, the technique is very robust and these assumptions need not be strongly adhered to. DA attempts to do this by forming one or more combinations of discriminating variables (i.e., discriminant functions) such as

(1) $$\begin{array}{c} D_{i}=d_{i1}Z_{i2}+d_{i2}Z_{2}+\cdots +d_{ip}Z_{p},\;i=1,\ldots ,n \end{array}$$

where *n* is the number of discriminant functions, *D*_*i*_ is the score on discriminant function *i*, the *d*'s are the weighting coefficients, and *Z*'s are the standard values of the *p* discriminating variables used in the analysis. That said, the functions, based on the weight of *d*'s coefficients, are formed in such a way as to maximize the separation between the groups and to minimize the distance within each group. Once the discriminant functions have been derived, the research objectives of DA, i.e., analysis and classification, are pursued [29].

In a second step the mean difference of variables selected by DA between each pair of manufacturers was examined; for more details see [30]. The multiple comparisons of mean difference between continuous variables were examined by using Dunnett's test. The level at which results were defined as being statistically significant was set at a P≤0.05. The calculations were performed using IBM SPSS Statistics, v.20.0 software (IBM Corp., Armonk, NY, USA).

## 3. Results and Discussion

**Figure 1:**
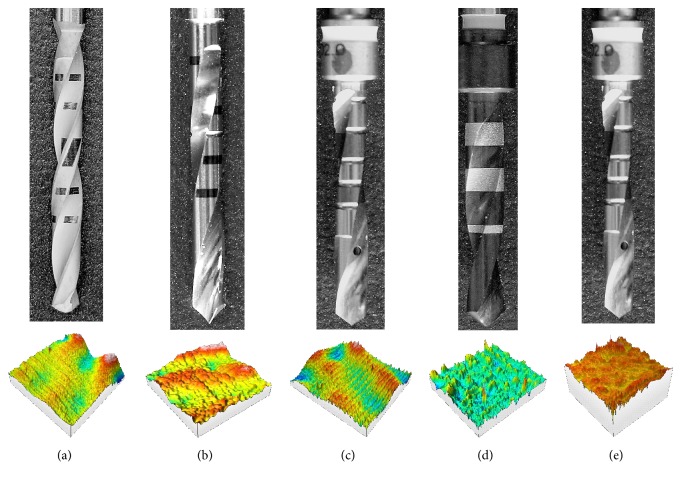
Top from left to right image of implant bone drill: (a), (b), (c), (d), (e); bottom corresponding reconstruction 3D of AoI.

*3.1.* The image of each bone drill and 3D details of the surfaces extracted from the five manufacturers' implant bone drills after leveling and filling points is shown in Figure 1.

The 3D surface reported below each bone drill represents the area taken from the surface that possesses minor isolated peaks, naturally present due to the complexity of the surface acquisitions by means of the confocal microscope. Therefore, to avoid as much irregularity as possible, a portion of the 500x500*μ*m^2^ of the acquired areas was considered. These Areas of Interest (AoI) have been chosen to perform as better as possible the comparative analysis among the bone drills considering, for a given drill, the surface portion that embraces part of its cylindrical external surface, near the cutting edges.

**Figure 2:**
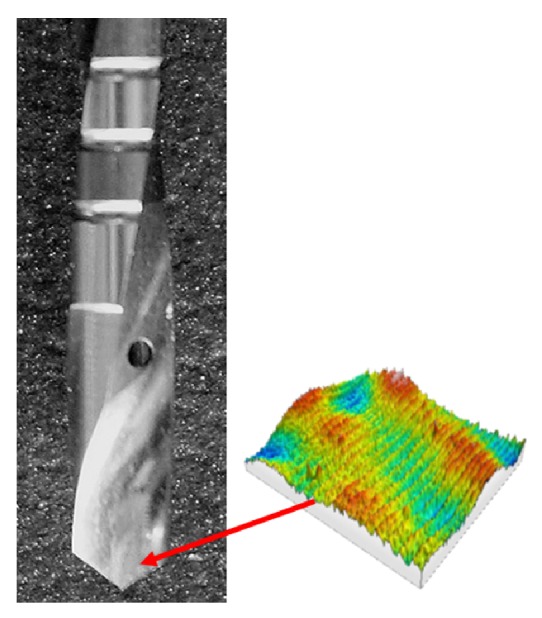
Close-up of AoI showing the location where the measurements were performed.

Naturally, comparative analysis has been performed taking into account every cutting edge for each drilling tool, which are at least two, to enrich the experimental data for the statistical analysis and to obtain more accurate results (Figure 2).

Preliminary, it should be noted that no significant differences were found for the various cut-offs along the transversal direction.

**Figure 3:**
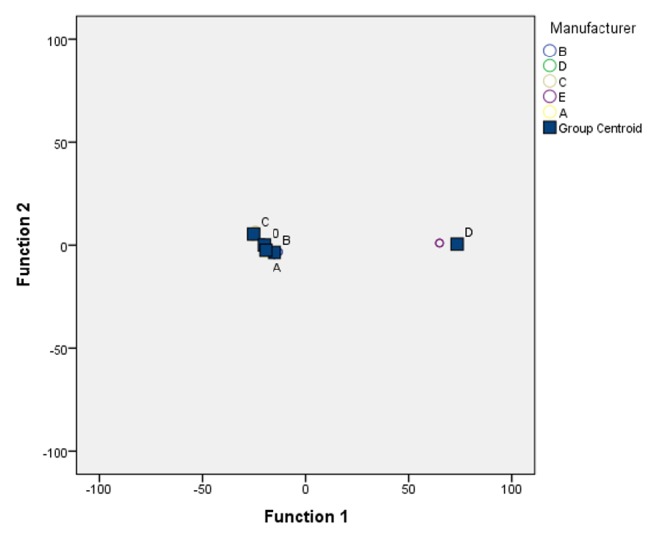
Cluster distribution of five manufacturers.

*3.2. *Robust Gaussian filter 0.08mm was used for the extraction of the roughness profiles parallel to the bone drill axis. The variables extracted through DA analysis ordered were as follows, according to the relative decreasing weight: R_q_, R_z_, R_mr_, R_ku_, P_sm_, R_sk_.  Figure 3 shows the cluster distribution of five manufacturers according to the canonical discriminant functions. In general, the closeness of the group centroids, marked with ■, to territorial lines suggests that the separation between all groups is not very strong. The centroids summarize the group locations in the “reduced” space defined by the discriminant functions.

The average values for each manufacturer and model parameter, for the cut-off = 0.08mm, are reported in Table 1. The last column reports the significant difference between manufacturers for each parameter.

In particular, R_q_ is defined as the root mean square deviation of the assessed profile. It corresponds to the standard deviation of the height distribution, defined on the sampling length. R_q_ provides the same information as R_a_ (arithmetic mean deviation of the assessed profile), but in a more accurate way since it is less sensitive to variations due to isolated peaks that affect the measure. The higher the dispersion, the greater the nonuniformity of the surface. Contextually, D represents the worst case, having the highest data dispersion. R_z_ parameter is the maximum height of the profile defined on the sampling length, evaluated as the mean distance from the 10 highest peaks and the 10 deepest valleys. Although being less accurate than R_q_, this parameter also represents a valid outcome variable, since it does not consider regular profiles with isolated peaks. Also, it is frequently used to check whether the profile has protruding peaks that might affect static or sliding contact function. In fact, this result has been confirmed from the values reported on the table, agreeing with the considerations made for R_q_. R_mr_, defined as the ratio of the material length Ml(*c*) of a profile curve element to the evaluation length at the sectioning level* c* (whether as % or *μ*m), enforces indications about the tailness of the profile, expressed as a ratio among vacancies and material confined in a defined virtual surface. In this case, more than a simple roughness indicator, this parameter offers an indication of the drill's integrity, and hence it is reasonable to take it into account in light of the evaluations explained herein. The previously defined R_sk_ and R_ku_ hybrid parameters represent the best indicators for the subject of this work: according to the definitions given above, it is preferable to have profiles with negative values of R_sk_, since they are characterized by a valley-shaped morphology, which is an advantage regarding heat generation and wear phenomena due to lower shear contacts with bones. In this context, bone drills corresponding to cases A, B, and E are the more preferable.

Different considerations have to be made for parameter R_ku_. It is well known that for values of this parameter less than 3, profile peaks are more likely to be round-shaped. Vice versa, when values are greater than 3, peaks are more likely to be pronounced. Standing on the results, cases C and E are the best since round-shaped edges of the bone drill's surfaces are preferable since they guarantee a longer tool life and a better quality of hole circularity, given that peaks can be more easily damaged during the cutting action. Naturally, this aspect is also related to the above-mentioned heat and wear phenomena.

*3.3. *Robust Gaussian filter 0.25mm was used for the extraction of roughness profiles parallel to the bone drill axis. The variables extracted through DA analysis are ordered according to the relative decreasing weight as follows: R_v_ and R_a_.

**Figure 4:**
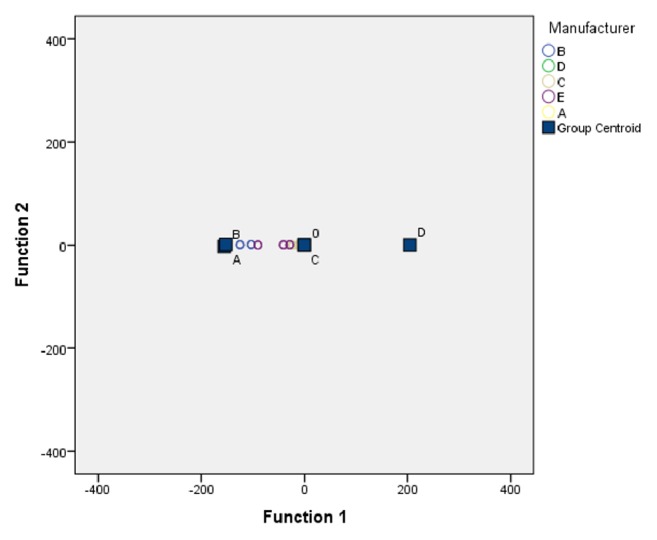
Cluster distribution of five manufacturers.

Figure 4 shows the cluster distribution of five manufacturers according to the canonical discriminant functions.

The average values for each manufacturer and model parameter and for the cut-off = 0.25mm are reported in Table 2. In the last column is reported the significant difference between manufacturers for each parameter.

As depicted in the table, R_a_ is highlighted as a key parameter. According to the discussion about the previous case, it is still a valid parameter, especially being the most widely used. This result also validates the goodness of the simulation methodology adopted. On the other hand, the R_v_ parameter deserves special consideration. More specifically, according to its definition as the maximum valley depth along a considered profile, it could not be considered as a global indicator of surface quality because it refers to isolated singularities, i.e., valleys. However, this result remains interesting since other tribological phenomena are related to this parameter, i.e., corrosion. In fact, the presence of a consistent number of deep valleys on a surface is more vulnerable to the effects of sterilization procedures [1, 12, 16], triggering localized corrosion phenomena like pitting. Based on these considerations, D represents again the worst case.

## 4. Conclusions

The study observed that the surface micromorphology, influencing the contact area between the drill and bone, can be considered as a factor that contributes to heat, wear, and corrosion phenomena due to material friction and corrosion resistance through several surface texture indicators. Not only the design, but also the micromorphology of the cutting surface directly contacting bone plays a key role in the cutting power and wear trend. The result of this research also showed considerable differences in the parameters examined in the implant drills present on the market, depending on the particular surface aspect to be analyzed in terms of its clinical impact.

## Figures and Tables

**Table 1:** Crosstab manufacturer versus mean model parameters, significant difference, cut-off 0.08mm.

**Manufacturer** **versus model parameter**	**A**	**B**	**C**	**D**	**E**	**Significant difference**
**Rq**	0.10	1.01	0.77	8.61	2.17	A-B, A-C, A-D, B-D, C-D, E-D
**Rz**	0.38	2.85	1.965	21.405	3.80	A-B, A-C, A-D, A-E, B-D, C-D, E-D
**Rmr**	99.00	33.27	4.17	1.37	1.53	A-B, A-C, A-D, A-E
**Rku**	2.73	3.00	1.97	3.43	1.73	A-E, B-E, C-D, D-E
**Psm**	28.00	76.67	190.00	53.67	101.50	A-B, A-C, A-E, B-C, C-D, C-E
**Rsk**	-0.05	-0.46	0.58	0.46	-0.18	A-C, A-D, B-C, B-D, C-E

**Table 2:** Crosstab manufacturer versus mean model parameters, significant difference, cut-off 0.25mm.

**Manufacturer** **versus ** **model parameter**	**A**	**B**	**C**	**D**	**E**	**Significant difference**
**Rv**	0.32	1.35	4.56	11.20	3.52	A-B, A-C, A-D, A-E, B-C, B-D, B-E, C-D, D-E
**Ra**	0.15	1.77	1.98	4.80	2.32	A-B, A-C, A-D, A-E, C-D, C-E, E-D
